# Primary Tumor Radiomic Model for Identifying Extrahepatic Metastasis of Hepatocellular Carcinoma Based on Contrast Enhanced Computed Tomography

**DOI:** 10.3390/diagnostics13010102

**Published:** 2022-12-29

**Authors:** Lawrence Wing Chi Chan, Sze Chuen Cesar Wong, William Chi Shing Cho, Mohan Huang, Fei Zhang, Man Lik Chui, Una Ngo Yin Lai, Tiffany Yuen Kwan Chan, Zoe Hoi Ching Cheung, Jerry Chun Yin Cheung, Kin Fu Tang, Man Long Tse, Hung Kit Wong, Hugo Man Fung Kwok, Xinping Shen, Sailong Zhang, Keith Wan Hang Chiu

**Affiliations:** 1 Department of Health Technology and Informatics, The Hong Kong Polytechnic University, Hong Kong SAR, China; 2 Department of Applied Biology and Chemical Technology, The Hong Kong Polytechnic University, Hong Kong SAR, China; 3 Department of Clinical Oncology, Queen Elizabeth Hospital, Hong Kong SAR, China; 4 Department of Radiology, The University of Hong Kong-Shenzhen Hospital, Shenzhen 518053, China; 5 Department of Diagnostic Radiology, The University of Hong Kong, Hong Kong SAR, China; 6 Department of Radiology & Imaging, Queen Elizabeth Hospital, Hong Kong SAR, China

**Keywords:** computed tomography, radiomics, machine learning, clinical decision-making, hepatocellular carcinoma, extrahepatic metastasis, oversampling

## Abstract

This study aimed to identify radiomic features of primary tumor and develop a model for indicating extrahepatic metastasis of hepatocellular carcinoma (HCC). Contrast-enhanced computed tomographic (CT) images of 177 HCC cases, including 26 metastatic (MET) and 151 non-metastatic (non-MET), were retrospectively collected and analyzed. For each case, 851 radiomic features, which quantify shape, intensity, texture, and heterogeneity within the segmented volume of the largest HCC tumor in arterial phase, were extracted using Pyradiomics. The dataset was randomly split into training and test sets. Synthetic Minority Oversampling Technique (SMOTE) was performed to augment the training set to 145 MET and 145 non-MET cases. The test set consists of six MET and six non-MET cases. The external validation set is comprised of 20 MET and 25 non-MET cases collected from an independent clinical unit. Logistic regression and support vector machine (SVM) models were identified based on the features selected using the stepwise forward method while the deep convolution neural network, visual geometry group 16 (VGG16), was trained using CT images directly. Grey-level size zone matrix (GLSZM) features constitute four of eight selected predictors of metastasis due to their perceptiveness to the tumor heterogeneity. The radiomic logistic regression model yielded an area under receiver operating characteristic curve (AUROC) of 0.944 on the test set and an AUROC of 0.744 on the external validation set. Logistic regression revealed no significant difference with SVM in the performance and outperformed VGG16 significantly. As extrahepatic metastasis workups, such as chest CT and bone scintigraphy, are standard but exhaustive, radiomic model facilitates a cost-effective method for stratifying HCC patients into eligibility groups of these workups.

## 1. Introduction

Hepatocellular carcinoma (HCC) is found to account for approximately 10% of cancer death worldwide, and it is particularly prevalent in Eastern and Southeastern Asian countries, including China [1]. The major risk factors of HCC are hepatic chronic diseases, especially the infection of hepatitis B virus and hepatitis C virus [2]. Despite improving prognosis by virtue of structured surveillance on known high-risk patients, HCC has very poor prognosis. Systemic therapy is conventionally used to treat patients with metastatic HCC and Sorafenib is the most commonly used first line treatment. However, it is not particularly well tolerated with treatment discontinuation due to side-effects in nearly 60% of patients [3]. Furthermore, as a small molecule multikinase inhibitor, both the VEGF receptor pathway and Raf kinase, critical to physiological function and homeostasis in many organs are blocked by Sorafenib. This can result in life-threatening complications, such as hemorrhage and cardiac events [4]. With locoregional therapies achieving excellent outcomes, it is paramount to accurately diagnose extra-hepatic disease in HCC patients.

Although extrahepatic metastasis is commonly known to be associated with intrahepatic masses with high heterogeneity in computed tomography and vascular invasion, these qualitative factors are relatively subjective as for individual radiologists. Thus, the dual-tracer positron emission tomography (PET)/CT is an emerging modality that has shown much promise due to its high reported sensitivity and accuracy for metastatic disease, but its availability is limited [5,6,7]. Without a promising approach for indicating extrahepatic metastasis clinically, the decision to obtain uniform metastasis workup is most likely made by the treatment provider [8].

While several biomarkers are reported to have a possible association with occurrence of metastasis of HCC, reliable biomarkers are yet to be standardized [9,10]. In past studies, quantitative image analysis is stated to be useful for deriving tumor dynamics in cellular and tissue level and developing imaging biomarkers, hence, could help to prognosticate underlying tumor biology [11,12]. At the same time, studies on radiomics of computed tomography (CT) of liver for predicting microvascular invasion (MVI), treatment outcome and recurrence of HCC are pointing to the possibility of radiomics in discovering imaging biomarkers related to HCC [12,13,14,15]. Prediction of MVI in 120 patients based on quantitative CT features yielded an area under the receiver-operating characteristic (ROC) curve (AUROC) of 0.80, positive predictive value of 63%, and negative predictive value of 85% [13]. A study proposed a radiomics Cox model for stratifying 88 patients with HCC treated with transarterial chemoembolization (TACE) into the high and low risk groups and compared its performance with the clinical score model [14]. Based on the proposed model, the hazard ratio (HR) of the high-risk group with reference to the low-risk group attained 7.42, which was higher than HR based on the clinical score model, 4.84 [14]. Early HCC recurrence prediction in 215 patients based on CT radiomics yielded an AUC of 0.817 (95% CI: 0.758–0.866), sensitivity of 0.794, and specificity of 0.699 [15]. The potential of imaging biomarkers in predicting extrahepatic metastasis of HCC could probably promote more efficient diagnosis and better treatment planning for the HCC patients.

Radiomics is a developing field of image analysis that utilizes multiple data-mining algorithms to collect image features that are not visible to the naked eye and integrate them to obtain information for prediction or prognosis [11,16]. By constructing models accordingly, it could perform successful prediction and evaluation in certain clinical tasks [16]. Multiple studies have reported clear correlations between CT radiomics and clinical outcomes, while a further combination with selected clinical factors could achieve higher accuracies and clinical benefits [12,13,14,15,16]. Despite the potential, the generalization of radiomics as a clinical indicator still requires numerous refinements and standardizations to allow clinicians to confidently implement radiomics in patient management [11,17]. From previous studies, CT radiomics has shown the potential to predict MVI in HCC and recurrence of HCC [13,15], whereas the metastatic rate was predicted by clinical features only [5,8]. As far as we know, no published study has assessed the association between CT radiomics of HCC tumor and its metastatic risk. A pilot study would provide ground for deeper exploration in this aspect.

Contrast-enhanced CT is preliminarily used for diagnosis and staging of HCC, which is currently irreplaceable standard-of-care modality in HCC management [18]. If the radiomic features related to extrahepatic metastasis could be mined from the CT images of liver, additional information can be obtained without extra radiation exposure on the potential metastatic sites. The purpose of this study is to investigate the possibility of using radiomics features, obtained from contrast-enhanced CT images of the liver by a computational approach, to identify extrahepatic metastasis in HCC patients.

## 2. Materials and Methods

Approval from the Research Ethics Committee of the affiliated institution has been obtained for this retrospective data analytical study (HKU/HA HKW IRB reference no.: UW 19-072; HKU-SZH IRB reference no.: [2019]324). In addition, all adopted methods in the study follow the Declaration of Helsinki as monitored by the Institutional Review Board of the clinical units.

### 2.1. Patient Characteristics and Imaging Criteria

**Figure 1:**
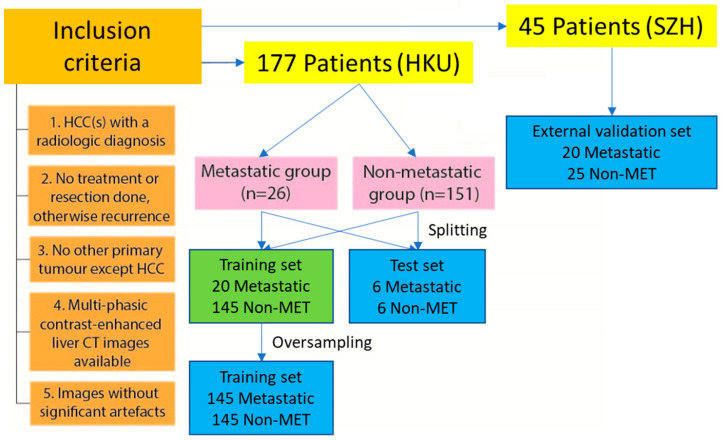
Flowchart of research case inclusion; Significant artefacts include breathing, moving, shunts or the iodized oils artefacts.

The retrospective data collected for this study initially consisted of the radiologist reports and CT images of 177 patients (mean age, 63.4 years; range, 31–83) from the image repository internally hosted by Department of Diagnostic Radiology, University of Hong Kong (HKU) and 45 patients (mean age, 55.4 years; range, 15–85) from University of Hong Kong-Shenzhen Hospital (SZH). To ensure confidentiality and privacy, all cases have undergone deidentification by the clinical staff who is not in research team before the data collection. The CT examinations were either requested for surveillance, diagnosis, or follow-up and were performed in the clinical unit between 2010 and 2019. The diagnosis of HCC was verified based on the radiologist reports and further confirmed by our supporting experienced radiologist (K.W.H.C.) according to guideline of American Association for the Study of Liver Diseases (AASLD)/Liver Imaging Reporting and Data System (LI-RADS) or European Association for the Study of the Liver (EASL). The inclusion criteria were HCC (s) with a radiologic diagnosis; multi-phasic contrast-enhanced liver CT images available. Exclusion criteria were treatment or resection conducted; recurrence; non-HCC primary tumor; significant artefacts including breathing, moving, shunts, or the iodized oils artefact arisen from the procedures of transjugular intrahepatic portosystemic shunt (TIPS), TACE or transcatheter oily chemoembolization (TOCE). Artefacts are regarded as significant when they visually distort the original appearance of HCC tumors. Due to the ghosting or smearing of the tumor region by breathing and motion, as well as the high attenuation of X-ray by the iodized oils and shunts near the tumors, untruthful pixel values within the tumor region would be resulted. Hence, these artefacts are regarded as significant. Inclusion of these artefacts would otherwise lead to inaccurate quantitative radiomics analysis. As shown in Figure 1, the cases collected from HKU were divided into metastatic group (n = 26; mean age, 61.6 years; range, 46–80) and non-metastatic group (n = 151; mean age, 63.7 years; range, 31–83), constituting the training and test sets. The cases collected from SZH were divided into metastatic group (n = 20; mean age, 55.4 years; range, 26–85) and non-metastatic group (n = 25; mean age, 55.32 years; range, 15–72), constituting the external validation set. Metastatic group was defined as reported metastasis to lymph node, lung, or any other regions except liver in the radiologist reports collected.

### 2.2. Training and Test Sets

The 177 cases are further divided into training and test sets by random split where six metastatic (MET) cases and six non-metastatic (non-MET) cases were randomly assigned to the test set. The remaining 20 metastatic cases and 145 non-metastatic cases were allocated in the training set. The demographic information and clinical characteristics of the training and test sets are compared in Table 1. No statistically significant difference in these parameters was found between the training and test sets.

### 2.3. Image Acquisition

Non-contrast and triple-phase CT images were acquired using 64-Multiple Detector CT (64-MDCT) scanners with a slice thickness of 1.25 mm in soft tissue window. The image acquisition of all cases was performed according to the same liver protocol, including the plain, arterial, portal venous, and delayed phase, with breath being held by the patients as instructed. The parameters of image acquisition protocol are shown in Table 2. The images at arterial phase were chosen for extraction of features. The arterial enhancement of HCC tumor allows accurate segmentation, i.e., the outlining of the HCC tumor, for further radiomics analysis [14].

### 2.4. Segmentation of HCC

**Figure 2:**
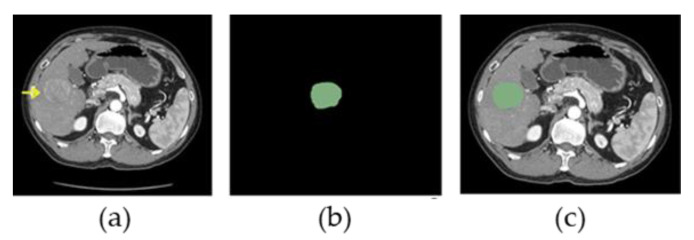
54-year-old man with hepatocellular carcinoma (HCC). (**a**) Original arterial phase CT scan slice shows the HCC lesion (yellow arrow). (**b**) ROI (green area) manually drawn in accordance with the HCC lesion. (**c**) Arterial phase CT scan slice shows an overlapped image of ROI (green area) and raw CT image.

Segmentation was performed on arterial phase CT images. It is important to note that radiomics has no restraint to any particular contrast-enhanced phase nor image modalities. Arterial phase was selected to undergo image analysis in this study because it has more radiological information on HCC compared to other phases [14]. Segmentation of the region of interest (ROI) of the largest primary HCC was performed by the research personnel with free segmentation software (Slicer 4.10.2). The contours were traced initially by trained operators and confirmed by an experienced radiologist (K.W.H.C.). An example of ROI segmentation is shown in Figure 2.

### 2.5. Feature Extraction

Radiomic features within the ROIs were computed using Pyradiomics, which is a Python package for quantitative radiographic analysis [19]. A set of 107 features was extracted from the original images according to 7 categories: 2D and 3D shape-based (14 features), first-order statistics (18 features), grey level cooccurrence matrix (GLCM) (24 features), grey level dependence matrix (GLDM) (14 features), grey level run length matrix (GLRLM) (16 features), grey level size zone matrix (GLSZM) (16 features), and neighboring grey tone difference matrix (NGTDM) (5 features). For additional feature extraction, images were further filtered by 8 Laplacian of Gaussian (LoG) wavelet transform filters and underwent the same algorithm. A set of 93 features were extracted (with shape-based features exempted) from each filtered image, and a total of 851 features was obtained for each tumor. Appendix A gives more detailed information of the 851 features.

### 2.6. Data Refinement

As the extracted features are distributed with different scales and significant diversity, it is necessary to transform them to a normal distribution with the same scale so that valid results could be obtained from logistic regression [20]. Rank-based inverse normal transformation was applied to every feature in the training, test and external validation sets separately [21].

Additionally, with the imbalanced data in the training set (20:145), the result would become biased and have low statistical significance [22]. In this regard, the synthetic minority oversampling technique (SMOTE) was applied to the training set to synthesize new examples for insufficient metastatic cases [22]. The SMOTE parameters, sampling strategy: ‘auto’, random state: ‘none’ and neighbors: 5, were used. A set of 125 new data samples were generated based on the existing samples so that the MET and non-MET groups of the training set have 145 cases each for analysis.

While 851 features were extracted and normalized to include the greatest extent of radiological information possible, the number of features was far more substantial compared to the number of the training cases, i.e., 290. Therefore, we need to sort out a more compact set of features for easier analysis and converged results with less redundancy and over-fitting [23]. We adopted a “feature ranking” approach to figure out the most essential and informative features in the training sets to be included in the model building. The feature ranking was performed by an automated univariate logistic regression in Python. All 851 features were automatically put through univariate forward regression one by one to obtain the prediction score for each feature based on the training set. The features were ranked by the prediction score in descending order, and the top 200 features were shortlisted for model building according to methods used by Aerts et al. (2014) [24]. The other feature selection methods were not applied in this study because the current approach is repeatable without specifying a random seed and the convergence to a unique model can be guaranteed.

### 2.7. Model Building and Statistical Analysis

Logistic regression is one of the most widely used machine learning algorithms that utilizes the supervised learning technique. We adopted stepwise forward binary logistic regression to determine features that can predict metastasis because logistic regression could produce more stabilized and reproducible results without fixing a particular random seed [25]. The patients are stratified into high or low risk of having extrahepatic metastasis by a logistic regression equation [26]:

Logit (Y_DM_) = b_0_ + b_1_X_1_ + b_2_X_2_ + … + b_n_X_n_, (1)

where X_n_ represents the value of the n^th^ radiomic features and b_n_ the corresponding coefficient related to the prediction of metastasis.

While logistic models are believed to have more consistent and less overfitting results with a ratio of 13 samples per predictor, we would select the top 8 features according to the training set of more than 124 cases (greater than 13 × 8) to construct the logistic regression model [27]. The performance of the model was indicated by sensitivity, specificity, AUC, and accuracy. Statistical analysis was performed with IBM SPSS Statistics 26. A *p*-value smaller than 0.05 indicated the effect or difference is statistically significant.

Deep learning and support vector machine (SVM) models were also trained using the training set and their performance metrics on the external validation set were compared with that of the logistic model. The statistical significance of difference in performance was determined by DeLong’s test for AUC and McNemar’s test for accuracy, sensitivity, and specificity.

## 3. Results

### 3.1. Patient Characteristics

The clinical features of 177 patients in the training and test sets are shown in Table 1 and Table 3. There were 151 metastatic cases and 26 non-metastatic cases. As shown in Table 3, cases with extrahepatic metastasis from HCC had statistically significantly larger tumor diameter (mean value: 5.10 cm vs. 8.06 cm, *p* = 0.007) and number of HCC lesions (mean value: 2.13 vs. 4.8, *p* = 0.011), while tumor diameter was also included as one of the radiomic features to be analyzed in the study. No statistically significant difference was found in other clinical features, such as hepatitis B and portal invasion (*p* > 0.05).

### 3.2. Training of Logistic Regression Model

**Figure 3:**
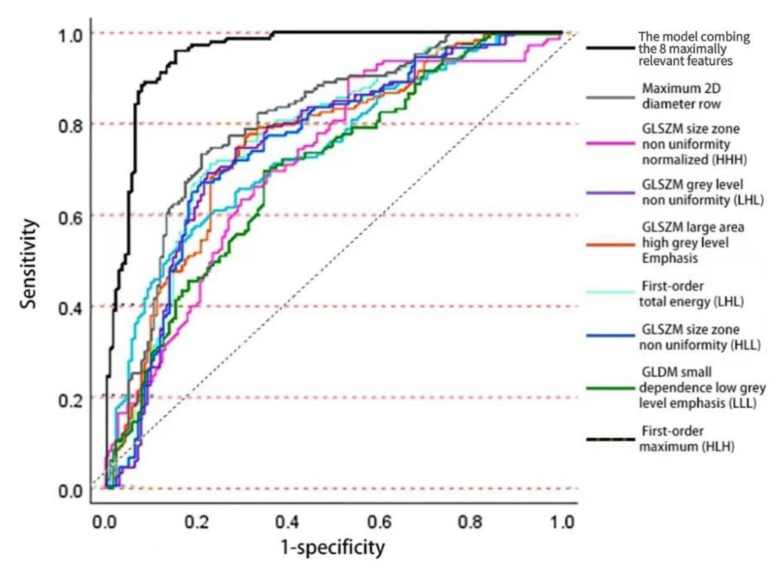
ROC curves of individual radiomic features and the logistic regression model of 8 selected features for identifying hepatocellular carcinoma (HCC) metastasis on the training set.

Among the top 200 features shortlisted from 851 extracted features, eight reproducible features were selected to form our predictive model in accordance with the logistic regression result (see Figure 3). The selected features, corresponding coefficients, and *p*-values of the trained logistic regression model are:

- maximum 2D diameter row (b_1_ = 2.371, *p* = 3.249 × 10^−8^),
- first-order total energy with wavelet LHL (b_2_ = 2.006, *p* = 0.067),
- first-order maximum with wavelet HLH (b_3_ = 0.476, *p* = 0.119),
- GLSZM size zone nonuniformity normalized with wavelet HHH (b_4_ = 0.986, *p* = 4.341 × 10^−6^),
- GLSZM grey level nonuniformity with wavelet LHL (b_5_ = −2.148, *p* = 0.050),
- GLSZM large area high grey level Emphasis in original image (b_6_ = 1.732, *p* = 0.024),
- GLSZM size zone nonuniformity with wavelet HLL (b_7_ = −2.001, *p* = 0.177), and
- GLDM small dependence low grey level emphasis with wavelet LLL (b_8_ = 1.439, *p* = 4.018 × 10^−3^).

The constant of logistic regression model is given by b_0_, −1.030 (*p* = 2.998 × 10^−7^).

### 3.3. Training of Deep Learning Model

Visual Geometry Group with 16 convolutional layers (VGG16) was used [28]. The training converged to optimal loss in 15 epochs with batch size of 18 and validation split of 0.2. The model attained an accuracy of 77.9%, with sensitivity of 77.9%, specificity of 80.7%, and AUC of 0.951 on the training set.

### 3.4. Training of SVM Model

SVM was used to identify the machine learning model. The input vector consists of eight radiomic features selected by logistic regression and linear kernel was used. With five-fold cross-validation, the hyperparameter C was optimized by searching seven grid points from −3 to 3. The model attained an accuracy of 90.0%, with sensitivity of 95.2%, specificity of 84.8%, and AUC of 0.938 on the training set.

### 3.5. Test and External Validation of Models

**Figure 4:**
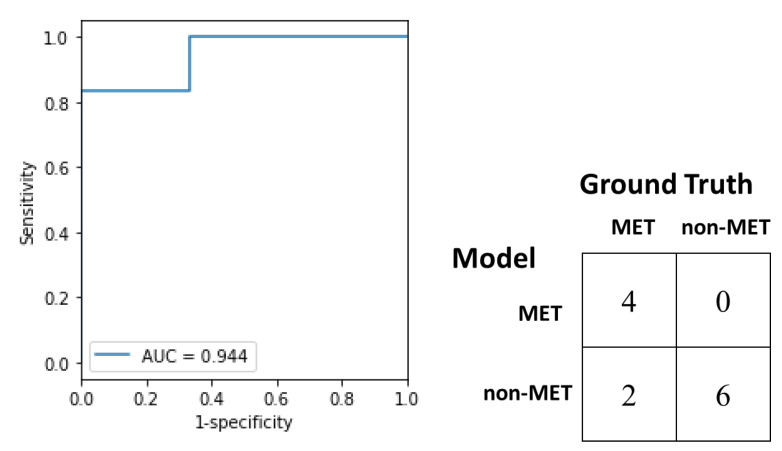
ROC curve and confusion matrix of the logistic regression model for identifying hepatocellular carcinoma (HCC) metastasis on the test set.

**Figure 5:**
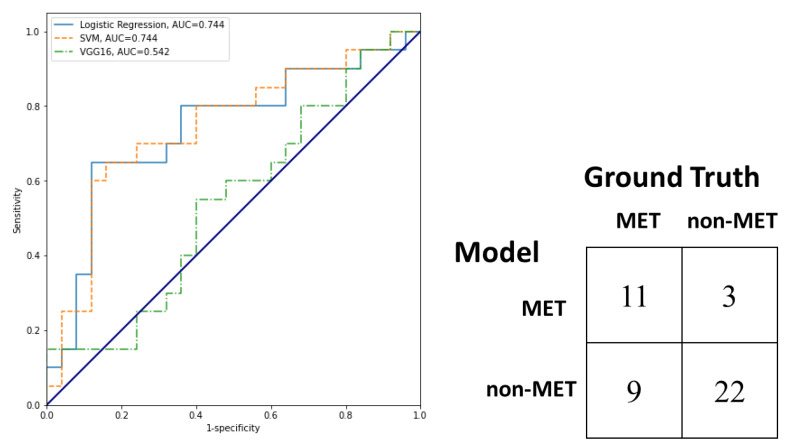
ROC curves of logistic regression, SVM and VGG16 model and confusion matrix of the logistic regression model for identifying hepatocellular carcinoma (HCC) metastasis on the external validation set.

On the test set, the trained logistic regression model attained accuracy of 83.3%, sensitivity of 66.6%, specificity of 100% and AUC of 0.944 (see Figure 4). On the external validation set, accuracy, sensitivity, specificity, balanced accuracy, F1, Matthews correlation coefficient (MCC), and AUC of logistic regression, SVM, and VGG16 were compared in Table 4 and the ROC curves were shown in Figure 5. Although there is no significant difference in AUC, accuracy, sensitivity, and specificity, all the performance metrics of logistic regression were higher than or equal to those of SVM. In contrast, logistic regression outperformed VGG16 significantly in terms of specificity (*p* = 0.021) and AUC (*p* = 0.044). Again, all the performance metrics of logistic regression were higher than or equal to those of VGG16. In the external validation, the logistic regression model attained accuracy of 73.3%, sensitivity of 55%, specificity of 88%, and AUC of 0.744.

## 4. Discussion

In the present study, we explored the possibility of using radiomics in contrast-enhanced CT to be a predictive indicator for metastasis disease in HCC patients. A radiomic model was constructed, and it showed its potential to individually identify HCC patients with high likelihood to have extrahepatic metastasis.

CT Imaging has become a crucial imaging modality in the management of HCC [18]. In recent years, the application of radiomics has allowed researchers to mine clinical and prognostic information from medical images by quantifying the phenotypic characteristics of tumors [16,29]. Various studies showed that CT images could predict the prognosis of HCC patients [12,13,14,15,16]. Detection of extrahepatic metastasis allows physicians to provide appropriate treatments for HCC patients although no previous study has explored the use of radiomics [6]. Thus, we designed this study to investigate possible predictors of extrahepatic metastasis, an important factor for patient prognosis and survival [5].

Radiomic features of different categories can quantify distinct intratumoral characteristics and thus reflect tumor complexity in multiple aspects. Despite the large number of features being tested, we further performed binary logistic regression and selected the first eight features of higher reproducibility and stability to avoid possible over-fitting of our model [25,26]. Half of the eight selected radiomic features were GLSZM based, one of them was shape based, one of them was GLDM based, and two of them were of first-order category.

GLSZM based features measure the spatial interrelationship of adjacent groups of grey level voxels in 13 directions three-dimensionally [30]. Four GLSZM features relevant to the nonuniformity of the grey level of the tumor in CT images were identified, indicating that the tumor heterogeneity was closely related to the possibility of metastasis. With generally higher magnitudes in GLSZM features of metastatic cases, the result can be related to the finding that textural heterogeneity in tumors could probably indicate metastasis, and hence poor prognosis and survival [12,31]. GLSZM based features have an advantage in that they are relatively more reproducible regardless of the segment accuracy and the interobserver reliability. Less precise segmentation could still generate similar results as the heterogeneity is often more significant in the center of the tumor but more subtle on the edges of ROIs [32].

Shape features quantify the shape and size of the ROI, including diameter, surface area and irregularity [30]. The selected shape feature measures the maximum axial diameter of the HCC drawn. Similar to the findings of Natsuizaka et al. [6], our results show that the longer the mean tumor diameter, the more likely the patient belonged to the metastatic group (*p* = 0.007).

GLDM based features mathematically describe the distributions of different grey levels within the ROI [33]. The small dependence low grey level emphasis measures the magnitude of low grey level distribution and indicates the density of voxels with low grey value in the ROI. We found that a smaller distribution of low grey value voxels may indicate higher likelihood of metastasis. This finding was consistent with the study by Mao, et al. [34], who found that less distribution of low grey level in ROI of arterial phase CT could be correlated to high-grade HCC, as it might reflect higher contrast enhancement and vascularity. High vascularity of HCC often promotes faster growth, infiltration, and invasion, thus increasing the likelihood of extrahepatic metastasis [5].

First-order features quantify the histogram distribution of the intensity values of the voxels in the ROI [35]. The two identified first-order features indicated that a histogram with higher total energy and maximum could stipulate extrahepatic metastasis. Kim et al. [14] reported a similar relationship between high energy in histogram and HCC tumor heterogeneity which could be related to metastasis, while a study by Peng, et al. [36] reported that a higher maximum in histogram could indicate microvascular invasion which directly increases the risk of extrahepatic metastasis. The first-order features we identified agreed with those in previous research.

On the external validation, the performance metrics of logistic regression were all comparable or better than SVM and VGG16. Significant difference in specificity and AUC between logistic regression and VGG16 was identified. VGG16 performed poorly because the relatively small training set was inadequate to train a very large network with huge number of weights. Although no significant difference between logistic regression and SVM was identified, the logistic regression yielded a more meaningful model where the coefficients represent the change in log odds of metastasis per unit change in the corresponding radiomic features. Based on logistic regression, the resultant radiomic model had AUCs of 0.914, 0.944, and 0.744 on the training, test and external validation sets respectively, which was comparable if not better than the performance of various similar radiomic models established by other researchers for predicting pathological or surgical outcomes of HCCs (AUCs: 0.670–0.859) [13,15,34,36,37,38]. The innovation of this study is that the radiomic model based on the image information of tumor region only can stratify the HCC patients into risk groups of extrahepatic metastases and support the decision for metastasis workups.

In the present study, we also identified some clinical features that might also have the capability to predict extrahepatic metastasis of HCC, including tumor diameter and number of lesions. While the clinical significance of tumor diameter was stated by various studies and was reflected in our radiomics model, the predictive power of number of lesions is controversial [5,6]. We performed univariate analysis on the HCC lesion numbers of cases and ranked it with the radiomic features extracted. The number of lesions was found to have less significant effect on extrahepatic metastasis when compared to the radiomic features. We also built a second model by combing the 8 selected radiomic features and the number of lesions and tested it using the same test set. The accuracy of the second model was not superior to our original model (accuracy: 75.0% vs. 83.3%). While our findings suggest that tumor numbers have limited predictive power for extrahepatic metastasis, studies by Uchino et al. [5] and by Natsuizaka et al. [6] reported it as an essential indicator for HCC metastasis in clinical practice. These contradictory conclusions might be resulted by various reasons. First, with a limited number of samples in our study, we might be unable to fully stratify metastatic and non-metastatic patients by a single clinical factor. Moreover, the number of tiny satellite lesions may not be completely reported in the radiologist reports, badly affecting the representing power of tumor numbers in our analysis. It is undeniable that number of lesions is a clinical feature that is far more accessible to the clinicians when compared to radiomic features, which might also be a reason for the tumor number to be a prognostic indicator for extrahepatic metastasis in hospital settings.

Our study has some limitations. The analytical results might be subjected to different standards in image acquisition, postprocessing and reconstruction across centers. Batch harmonization techniques, such as global scaling and z-standardization, were proposed to minimize feature variabilities [39]. A thorough assessment of the most appropriate technique is required for developing a radiomic model involving multiple centers. The study was also limited by a small sample size that could lead to instability in extraction and analysis of radiomic features, while the imbalanced data set might also cause inaccuracies in feature selection and analysis, although it has undergone SMOTE. Future studies with more comprehensive and larger samples are required to further verify our findings. We only extracted radiomic features from the largest HCC lesion in each case, as there were satellite lesions that were difficult to draw and might be subjected to measurement error [40]. The CT images were acquired by several different CT scanners over a few years of time. Differences between CT scanners, a change in protocols, use of different contrasts, and evolved reconstruction and postprocessing techniques might affect the radiomic features. Although the effects could be unintentionally reflecting the clinical reality that multiple CT scanners and protocols might be used clinically, it is still one of the limitations of our research design [14,40]. Since additional information, such as histological features, were not quite considered in the present study, future studies are needed to further interpret the radiomic features with biological markers. We believe that the modification of the model into a cluster-based search algorithm will allow clinicians to retrieve cases with similar radiomics features and clinical metastatic factors. Then, the model can assist clinicians in determining the MET possibility of a newly registered HCC case and suggesting which organs are at a higher MET risk.

## 5. Conclusions

Contrast-enhanced computed tomography (CECT) is commonly used for the diagnosis and staging of HCC to guide treatment options. However, certain information which are the clues of metastasis might not be perceived by human eyes. Compared with the traditional ways of confirming extrahepatic metastasis, including chest CT and bone scintigraphy, which entail exhaustiveness, higher cost, and limited availability, radiomics play an important role by allowing efficient quantification of multiple features extracted from arterial phase of liver CT images which are clinically significant but beyond human perception to detect extrahepatic metastasis in HCC patients. The developed model with top eight features selected with higher relevance and reproducibility in this study has shown its potential to perform better prediction than reported clinical features of metastasis of HCC and other existing radiomics models. Our findings could be useful for predicting the pathological status of HCC. Thus, this could possibly eliminate the need for extra metastatic scanning with radionuclide imaging to aid more timely decision-making to facilitate early targeted therapy and reduce unnecessary locoregional therapy for patients with extrahepatic metastasis. As a result, the model has shown its potential to increase patients’ survival rate while lowering patient anxiety and medical burdens.

## Figures and Tables

**Table 1:** Demographics and characteristics of 177 HCC cases in training and test sets.

Characteristics	Training Cohort (n = 165)	Test Set (n = 12)	*p* ^1^
Mean age	63.4	63.8	0.894
Sex			0.758
Male	130	9	
Female	35	3	
Hepatitis B	79	5	0.677
Liver cirrhosis	79	6	0.887
Mean tumor diameter (largest lesion, cm)	5.44	6.94	0.380
Mean number of HCC lesions	2.44	3.83	0.204
Portal invasion	35	3	0.758

^1^ *p*-value for independent group comparison indicates that there is not any subject assignment bias.

**Table 2:** Image acquisition protocol of MDCT examinations.

Protocol Item	Parameter
Scan parameters	
Peak kilo voltage output	120 kV
X-ray tube current	700 mA
Contrast medium injection parameters	
Contrast agent	Iopamidol/Iohexol
Concentration	350–370 mg/mL
Volume	100–120 mL
Flow rate	3–5 mL/s

**Table 3:** Comparison of demographic information and clinical characteristics between metastatic and non-metastatic groups.

Characteristics	No Metastasis (n = 151)	Metastasis (n = 26)	*p* ^1^
Mean age	63.7	61.6	0.353
Sex			0.413
Male	117	22	
Female	34	4	
Hepatitis B	73	11	0.569
Liver cirrhosis	76	9	0.138
Mean tumor diameter (largest lesion, cm)	5.10	8.06	0.007 *
Mean number of HCC lesions	2.13	4.80	0.011 *
Portal invasion	29	9	0.077

^1^ *p*-value for independent group comparison indicating that there is not any subject assignment bias, except mean tumor diameter and mean number of HCC lesions. * indicates statistically significant.

**Table 4:** Performance of logistic regression, SVM and VGG16 on external validation set.

Performance	Logistic	SVM	VGG16	Logit vs. SVM	Logit vs. VGG16
Accuracy	0.733	0.666	0.533	*p* = 0.223	*p* = 0.066
Sensitivity	0.55	0.40	0.50	*p* = 0.083	*p* = 0.763
Specificity	0.88	0.88	0.56	*p* = 1.000	*p* = 0.021
Balanced accuracy	0.715	0.640	0.544		
F1	0.647	0.516	0.539		
MCC	0.462	0.324	0.089		
AUC	0.744	0.744	0.542	*p* = 0.905	*p* = 0.044

## Data Availability

Not applicable.

## Appendix A

The following radiomic features were extracted:

**Table A1:** From original image only.

Shape	Elongation
(14 features)	Flatness
	Least axis length
	Major axis length
	Maximum 2D diameter column
	Maximum 2D diameter row
	Maximum 2D diameter slice
	Maximum 3D diameter
	Mesh volume
	Minor axis length
	Sphericity
	Surface area
	Surface volume ratio
	Voxel volume

**Table A2:** From original image and images filtered by 8 wavelet transform filters (HHH, HHL, HLH, HLL, LHH, LHL, LLH, LLL).

First order	10 percentiles
(18 features)	90 percentiles
	Energy
	Entropy
	Interquartile range
	Kurtosis
	Maximum
	Mean absolute deviation
	Mean
	Median
	Minimum
	Range
	Robust mean absolute deviation
	Root mean squared
	Skewness
	Total energy
	Uniformity
	Variance
GLCM	Autocorrelation
(24 features)	Cluster prominence
	Cluster shade
	Cluster tendency
	Contrast
	Correlation
	Difference average
	Difference entropy
	Difference variance
	Inverse difference
	Inverse difference moment
	Inverse difference moment normalised
	Inverse difference normalised
	Informational measure of correlation 1
	Informational measure of correlation 2
	Inverse variance
	Joint average
	Joint energy
	Joint entropy
	Maximal correlation coefficient
	Maximum probability
	Sum average
	Sum entropy
	Sum squares
GLDM	Dependence entropy
(14 features)	Dependence nonuniformity
	Dependence nonuniformity normalised
	Dependence variance
	Grey level nonuniformity
	Grey level variance
	High grey level emphasis
	Large dependence emphasis
	Large dependence high grey level emphasis
	Large dependence low grey level emphasis
	Low grey level emphasis
	Small dependence emphasis
	Small dependence high grey level emphasis
	Small dependence low grey level emphasis
GLRLM	Grey level nonuniformity
(16 features)	Grey level nonuniformity normalised
	Grey level variance
	High grey level run emphasis
	Long run emphasis
	Long run high grey level emphasis
	Long run low grey level emphasis
	Low grey level emphasis
	Run entropy
	Run length nonuniformity
	Run length nonuniformity normalised
	Run percentage
	Run variance
	Short run emphasis
	Short run high grey level emphasis
	Short run low grey level emphasis
GLSZM	Grey level nonuniformity
(16 features)	Grey level nonuniformity normalised
	Grey level variance
	High grey zone emphasis
	Large area emphasis
	Large area high grey level emphasis
	Large area low grey level emphasis
	Low grey level zone emphasis
	Size zone nonuniformity
	Size zone nonuniformity normalised
	Small area emphasis
	Small area high grey level emphasis
	Small area low grey level emphasis
	Zone entropy
	Zone percentage
	Zone variance
NGTDM	Busyness
(5 features)	Coarseness
	Complexity
	Contrast
	Strength
